# Differential contribution of α2δ auxiliary subunits of voltage-gated calcium channels in mouse models of pain and itch

**DOI:** 10.1371/journal.pone.0337701

**Published:** 2025-12-02

**Authors:** Joao M. Braz, Madison Jewell, Karnika Bhardwaj, Sian Rodriguez-Rosado, Veronica Craik, Allan I. Basbaum

**Affiliations:** Department of Anatomy, University of California, San Francisco, United States of America; Indiana University School of Medicine, UNITED STATES OF AMERICA

## Abstract

Voltage-gated calcium channels (VGCCs) are multimeric proteins composed of alpha 1, β and γ subunits, as well as one of four auxiliary α2δ subunits. Although there is considerable preclinical and clinical evidence for a contribution of VGCCs to nociceptive processing, notably the gabapentin-targeted α2δ-1 subunit, unclear is the extent to which other α2δ subunits contribute to baseline or injury-altered pain and itch processing. Here, we investigated the anatomical and behavioral consequences of deleting α2δ-2, α2δ-3 or α2δ-4 in the mouse and report that selectively ablating each α2δ subunit leads to different, and in some cases, opposite effects on behavioral indices of pain and itch. Specifically, deleting α2δ2 resulted in mechanical and heat hypersensitivity, and an increase in spinal cord microglial immunoreactivity, but reduced scratching (presumptive) itch in response to a pruritogen. In contrast, ablation of α2δ3 led to thermal hyposensitivity, but no change in mechanical responsiveness or indices of itch. Mice deficient for α2δ4 exhibited hyposensitivity across pain modalities and only minor itch deficits. Interestingly, these differential effects were limited to baseline nociceptive responses, therefore we conclude that the α2δ-2, α2δ-3 and α2δ-4 subunits of VGCCs differentially contribute to pain and itch processing. The mechanisms underlying these differences remain however to be determined.

## Introduction

Voltage-gated calcium channels (VGCC) are heteromultimeric protein complexes that allow calcium influx upon membrane depolarization and therefore control key calcium-dependent intracellular processes, including neuronal excitability, neurotransmitter release as well as gene transcription [1]. VGCCs are composed of multiple subunits that include a pore-forming α1 subunit as well as auxiliary α2δ, β, and γ subunits, which have a wide range of functions [2,3] including assembly and axonal trafficking of the channel complex [4], interaction with intracellular and synaptic partners [5,6], excitatory synapse formation, as well as synaptic transmission [7,8]. Not surprisingly, therefore, mutations in VGCC auxiliary subunits are associated with many human neurological conditions, including retinal and auditory disorders, ataxia and epilepsy [9].

VGCCs are major contributors to nociceptive processing [10–13]. Here we focus on the contribution of the four auxiliary α2δ subunits, which are encoded by 4 distinct genes (*Cacna2d1-4*) and are differentially distributed in primary sensory neurons, spinal cord and brain [14]. The α2δ-1 and, to some extent, α2δ-2 subunits are the therapeutic targets of gabapentinoids, the first line pharmacological approach to the management of neuropathic pain [15–17]. Consistent with a contribution to “pain” transmission, preclinical studies in mice reported that genetic deletion of α2δ-1 reduces mechanical and cold, but not thermal (heat) responses [11] and delays the development of peripheral nerve injury-induced mechanical allodynia. Mice deficient in the α2δ-3 subunit exhibit reduced heat “pain” responses as well as a delay in thermal sensitization in a model of inflammatory pain [18,19]. Genetic deletion of α2δ-2 provokes cerebellar ataxia and seizures in mice [20,21], but to our knowledge, there are no preclinical studies of the consequence of α2δ-2 deletion on nociceptive processing. Although not directly tested in models of acute or chronic pain, α2δ-4 mutant mice are hyperactive and exhibit anxiolytic and anti-depressive behaviors [22], which may have relevance in chronic pain settings.

Here we first examined the extent to which α2δ-2, α2δ-3 or α2δ-4 subunits contribute to different modalities of pain, at baseline, in naïve conditions and in different chronic pain models. We integrated an extensive anatomical and behavioral analysis in wild type mice and in mice deficient in the α2δ-2, α2δ-3 or α2δ-4 auxiliary subunits. In addition to characterizing the expression pattern of these subunits in primary sensory and spinal cord neurons, we evaluated the behavioral responses of mice in a battery of mechanical, heat and cold pain tests, before and after tissue (inflammatory pain) or peripheral nerve (neuropathic pain) injury. To assess the contribution of these subunits to the generation of itch, we also examined their responses to a nape of the neck injection of pruritogens. We report that selectively ablating each α2δ subunit leads to different, and in some cases, opposite effects on baseline indices of pain and itch. The mechanisms underlying these differences remain however to be determined.

## Materials and methods

### Animals

All animal experiments were approved by the UCSF Institutional Animal Care and Use Committee and conducted in accordance with the NIH Guide for the Care and Use of Laboratory animals. Animals were euthanized at the end of the experimental endpoints or when they reached humane endpoints, using a chemical method (CO_2_ inhalation) followed by a physical method (cervical dislocation), as per animal protocol #AN199730. Animals used for *in situ* hybridization were euthanized with an overdose of Avertin (Tribromoethanol, 250 mg/kg), followed by bilateral thoracotomy and intracardial perfusion. There were no unexpected adverse events observed during the course of this study and peri-operative analgesia and anesthesia were performed, according to animal protocol #AN199730 to minimize stress and pain. Heterozygous mice were purchased from Jackson (Cacna2d4, strain #035183) or UC Davis (Cacna2d2 [stock #066863-UCD] and Cacna2d3(tm1a) [stock #046869-UCD]) and we generated homozygous mice (complete KO), in house, for subsequent behavioral analyses. Conditional (floxed) Cacna2d3 knockout mice were purchased from UC Davis [Cacna2d3(tm1c); stock #046869-UCD] and were crossed with mice that express the Cre recombinase selectively in dorsal root ganglion (DRG) neurons (Advillin-Cre; kindly provided by Dr. Zhonghui Guan at UCSF [23]), which generated double transgenic fl.*Cacna2d3* x *Advillin*-Cre mice. Mice were housed in cages on a standard 12:12 hour light/dark cycle with food and water ad libitum. Both male and female mice were used for behavioral testing.

### Behavioral analyses

For all behavioral tests, the mice were first habituated for 1 hour in Plexiglas cylinders. All mechanical, thermal, motor and pruritogen tests were performed as described previously [24]. Briefly, for the Hargreaves test of heat pain sensitivity, we measured latency to withdraw the hind paw from a heat source that was applied through a glass surface. For the hot plate test (50, 52 and 55°C), which provides an integrated measure of the supraspinal as well as spinal cord contribution to pain processing, we recorded the latency to lick or flinch the hind paws or to jump. To measure mechanical paw thresholds, we placed mice into clear plastic chambers on a wire mesh grid and stimulated the hind paw with graded von Frey filaments. We used the up-down method [25] to measure mechanical withdrawal thresholds. For cold responsiveness, we applied a drop of acetone (~25µl) to the plantar surface of the hind paw 5 times every 30 sec and then recorded the number of nocifensive behaviors (number of paw lifts/licks/shakes/bites), over the 5 applications. Motor coordination was evaluated using the rotarod test. The experimenter performing the behavioral testing was always blind to the different groups and the same experimenter tested both male and female mice of the same line. However, as different experimenters tested different knockout mouse lines, baseline thresholds may vary across different groups. For this reason, and given the low sample of certain groups, we only compared WT vs KO, within each sex of each genotype, and not across sexes.

### Statistics

All statistical analyses were performed with Prism (Graph Pad) and data are reported as mean + /- SD. Student *t*-tests and Mann-Whitney tests were used for single comparisons between two groups. As there was no significant difference in the rotarod test between WT and KO mice, regardless of sex, the data for male and female mice were combined in the same histogram.

### Pruritogen-evoked scratching

One day prior to testing, the mice were shaved at the nape of the neck. The following day, a pruritogen, (histamine (500 μg; Sigma) or chloroquine (100 μg; Sigma), both dissolved in saline), was injected subcutaneously (100 μl) into the neck. We video recorded the injected mice and counted scratching bouts that occurred during the first 30 minutes after the injection.

### Spared-nerve injury (SNI) model of neuropathic pain

The SNI model was produced as described previously [26]. Briefly, under isoflurane anesthesia, we ligated and transected two of the three branches of the sciatic nerve, sparing the tibial nerve. We tested the mechanical thresholds before (baseline) and 7 days after injury. Post injury heat thresholds were not tested as heat hypersensitivity is not commonly produced with this model.

### Inflammatory pain model

To induce chronic inflammation, mice were injected with Complete Freund’s Adjuvant (CFA; Sigma). CFA was diluted 1:1 with saline and vortexed for 30 min. When fully suspended, we injected 20 μL of CFA into the plantar surface of the mouse. Withdrawal thresholds were measured before the injection (baseline) as well as 3 days after, using the Hargreaves test.

### Immunohistochemistry

Mice were anesthetized with an overdose of Avertin (250 mg/kg) and then perfused with 10 mL of saline followed by 30 mL of 3.7% formaldehyde. Tissues were dissected out, postfixed, cryoprotected and then cut on a cryostat. Lumbar spinal cord (25µm) sections were preincubated for 30 minutes at room temperature (RT) in Tris-phosphate-buffered saline (PBST; 0.5% Triton X-100, 10% BSA, and 10% normal goat serum) and then immunostained overnight at RT in 10% PBST containing the primary antibody (rabbit anti-Iba1, 1:1000, Wako, cat. #019–19741). After washing in PBS, sections were incubated for 1 hour with a secondary antibody (Alexa 546-conjugated IgG; 1:1000, Millipore), rinsed in PBS, mounted in Fluoromount-G (Southern Biotechnology, Birmingham, AL), and coverslipped.

### *In situ* hybridization (ISH)

We followed the Advanced Cell Diagnostics protocol for RNAscope ISH (Advanced Cell Diagnostics, multiplex fluorescent assay, cat. #320850). *In situ* hybridization was performed, as previously described [27], using fresh lumbar dorsal root ganglia (DRG) and spinal cord tissue from adult male mice (8–10 weeks old).

### Imaging

All images were collected on an LSM 700 confocal microscope (Zeiss) equipped with 405-nm (5-mW fiber output), 488-nm (10-mW fiber output), 555-nm (10-mW fiber output), and 639-nm (5-mW fiber output) diode lasers and a 20x Plan-Apochromat (20x/0.8) objective (Zeiss). Image acquisition was performed with ZEN 2010 (Zeiss). Image dimensions were 1,024 × 1,024 pixels with an image depth of 12 bits. Two-times averaging was applied during image acquisition. To avoid saturation of single pixels, the laser power and gain were adjusted, and we used Fiji/ImageJ to control for brightness/contrast and assigning of artificial colors. The same imaging parameters and adjustments were used for all images within an experiment.

For quantitative analysis of transcript expression, we analyzed 12μm (DRG) and 25μm (spinal cord) sections from 3–4 mice per group (5–6 sections per tissue).

### Cell counts

To analyze overlap of probes by *in situ* hybridization, we first counted the number of neurons in the DRG that were positive for the Cacna2d subunit and then determined the percentage of Cacna2d+ neurons that were double labeled with the second probe. The percentage of double-labeled neurons (marker vs Cacna2d+) was calculated by dividing the number of double-labeled neurons by the number of single Cacna2d-labeled neuron x 100. To conclude that cells were double-labeled by *in situ* hybridization, we set a threshold of at least five fluorescent ‘dots’ for each probe in conjunction with a DAPI-positive nucleus.

### Intensity measurement (whole image)

To quantify the expression (intensity) levels of each Cacna2d subunit, before and after tissue (CFA) or nerve (SNI) injury, we processed the *in situ* images with ImageJ software [Mean intensity fluorescence = integrative density – (area x mean background)], using 8 sections per mouse and 3 mice per group. Intensity background was determined according to the specifications of the software, namely by selecting a region, within each image, with no fluorescence. Values are presented as mean ± standard deviation (SD). Statistical significance was assessed with a Student’s t-test. A p-value of 0.05 was considered significant and is indicated with an asterisk (*).

## Results

### 1) α2δ auxiliary subunit mRNA expression in DRG and spinal cord neurons

We first investigated the expression pattern of the 3 auxiliary subunits: α2δ-2 (*Cacna2d2* gene), α2δ-3 (*Cacna2d3* gene) and α2δ-4 (*Cacna2d4* gene) in DRG and spinal cord neurons, before and 7 days after partial peripheral nerve injury or 3 days after CFA-induced inflammation of the hind paw. For comparison, we also analyzed expression of the well characterized subunit α2δ-1 (*Cacna2d1* gene), which has been implicated in neuropathic pain and is the target of gabapentin [15–17].

Using multiple labeling *in situ* hybridization, we first analyzed the distribution pattern of each subunit in DRG neurons and included gene markers that are selectively expressed by subsets of sensory neurons [28]. To mark neurons with myelinated axons, we monitored *Nefh* expression, which encodes the neurofilament NF200; for unmyelinated peptidergic neurons, we monitored *Tac1* expression, which encodes the peptide substance P; for unmyelinated nonpeptidergic neurons, we monitored expression of *PAP*, which encodes the prostatic acid phosphatase enzyme [29]. Lastly, to mark C-low threshold mechanoreceptors (C-LTMRs), we monitored expression of tyrosine hydroxylase (TH) [30].

**Fig 1:**
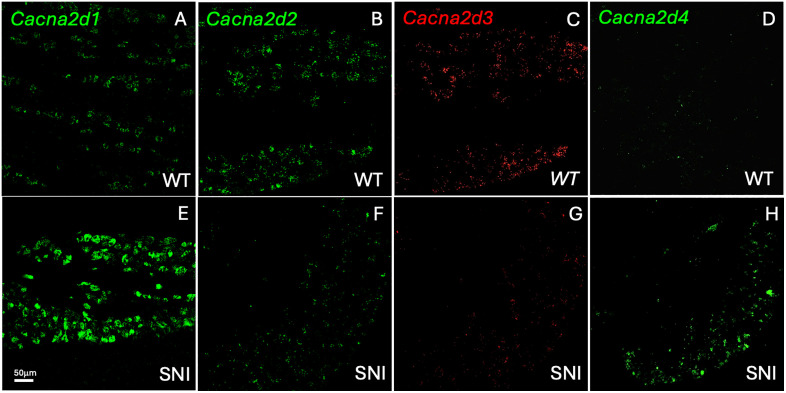
DRG expression of VGCCs is modulated by peripheral nerve injury. *In situ* hybridization using selective probes for *Cacna2d1* (A, E), C*acna2d2* (B, F), *Cacna2d3* (C, G) and *Cacna2d4* (D, H) revealed that the expression of *Cacna2d1* and *Cacna2d4* is upregulated in lumbar sensory neurons 7 days following a peripheral nerve injury (SNI) whereas *Cacna2d2* and *Cacna2d3* expression is downregulated.

**Fig 2:**
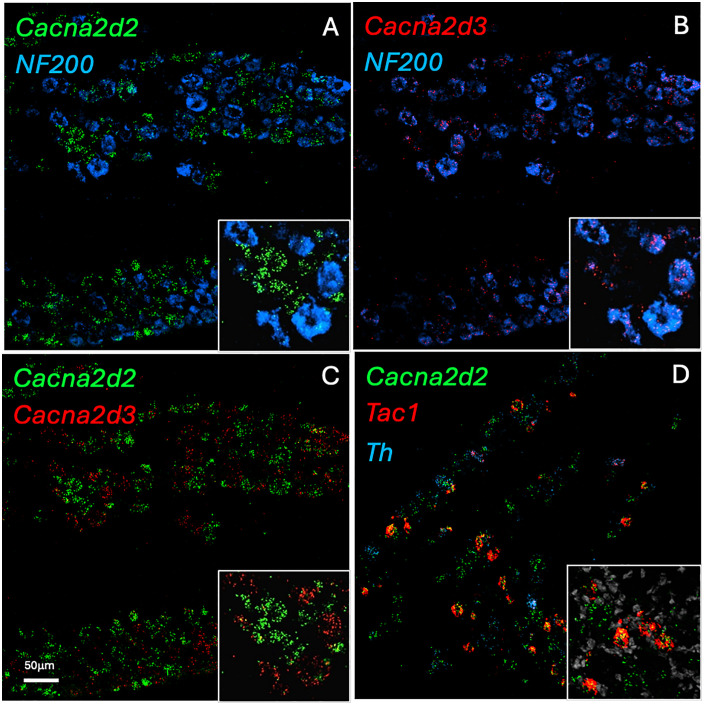
DRG expression pattern of VGCC subtypes. *In situ* hybridization revealed that whereas *Cacna2d2* (A; green) is predominantly expressed in unmyelinated (NF200 negative; blue) sensory neurons, *Cacna2d3* (B; red) is mainly found in myelinated neurons. (C) Thus, *Cacna2d2* (green) and *Cacna2d3* (red) are expressed in non-overlapping subsets. (D) Within the unmyelinated subset, *Cacna2d2* can be found in neurons expressing Substance P (*Tac1*; red) as well as those expressing Tyrosine hydroxylase (*Th*; blue), a marker of C-LTMRs.

As previously reported, *Cacna2d1* is expressed in a large and heterogeneous population of DRG neurons (Fig 1), including both myelinated (33.9 ± 6.2% Nefh + ; N = 3) and unmyelinated (40.6 ± 12.7% PAP + ; N = 3) neurons. In contrast, *Cacna2d2* and *Cacna2d3* are largely expressed in non-overlapping subsets (Figs 1B-C and Fig 2). Specifically, *Cacna2d3* predominates in myelinated (65.8 ± 2.3% Nefh + ; N = 3) neurons, consistent with the results of Usoskin et al., whereas *Cacna2d2* expression predominates in unmyelinated sensory neurons, as only 37.9 ± 1.5% overlapped with Nefh (N = 3). Consistent with its expression in the unmyelinated subset, we found that 31.1 ± 0.4% of *Cacna2d2* expressed the peptidergic marker *Tac1* (N = 3) and 11.2 ± 1.2% were expressed in C-LTMRs (TH + ; N = 3). On the other hand, *Cacna2d4* mRNA expression was very low in DRG neurons at baseline (Fig 1D). We also detected mRNA expression of all subunits in both the dorsal and ventral horns of the spinal cord (S1 Fig and Table 1), except for *Cacna2d4*, for which levels were very low to undetectable.

**Table 1:** Modulation of the α2δ expression levels by tissue or nerve injury. The expression levels of each VGCC auxiliary α2δ subtype was quantified using *in situ* hybridization in naïve (WT) mice and 7 days (SNI) or 3 days (CFA) after nerve (SNI) or tissue (CFA) injury, respectively. Values represent mean intensity fluorescence (N = 3; levels within each tissue were compared between WT and SNI or CFA using Student’s t-test * p < 0.05; ** p < 0.01, *** p < 0.005).

Gene (N = 3)	DRG (WT)	DRG (SNI; 7d)	SpC-DH (WT)	SpC-DH (SNI; 7d)	SpC-VH (WT)	SpC-VH (SNI; 7d)	DRG (WT)	DRG (CFA; 3d)
Cacna2d1	125.7 ± 30.2	487.2 ± 36.5***	17.9 ± 1.3	26.9 ± 1.8*	1.9 ± 1.0	7.2 ± 1.7*	41.9 ± 9.9	55.7 ± 13.9
Cacna2d2	40.9 ± 5.9	20.8 ± 3.7*	70.7 ± 22.6	72.7 ± 7.1	65.2 ± 8.7	49.9 ± 21.7	44.1 ± 7.5	45.5 ± 6.0
Cacna2d3	23.2 ± 1.3	13.0 ± 3.4*	95.4 ± 31.5	124.9 ± 50.7	162.1 ± 34.2	155.7 ± 25.0	33.4 ± 10.0	21.6 ± 2.2
Cacna2d4	8.2 ± 1.8	23.9 ± 5.0 *	22.4 ± 6.3	16.2 ± 7.3	16.9 ± 7.0	14.8 ± 2.9	15.4 ± 3.0	10.7 ± 0.8

Next, we investigated whether expression of the subunits is modulated by tissue or nerve injury. Consistent with earlier reports [31,32], the expression of *Cacna2d1* in both DRG and spinal cord increases significantly after partial sciatic nerve injury (SNI). In distinct contrast, the expression levels of *Cacna2d2* and *Cacna2d3* decreased significantly in DRG neurons (Fig 1F-G and Table 1). Most interestingly, the expression of *Cacna2d4,* which was low to undetectable in naïve mice, was significantly induced in DRG after SNI (Fig 1H), including in many peripherin-positive, presumptive nociceptors (54.2 ± 1.1% Per+; N = 3). In contrast to the post SNI changes in sensory neurons, except for a significant increase in *Cacna2d1* expression in both dorsal and ventral horns of the spinal cord, we did not detect significant changes in spinal cord expression of α2δ-2, α2δ-3 or α2δ-4. CFA-induced inflammation also did not significantly alter the expression levels of any of the subunits in sensory neurons (Table 1).

### 2) Behavioral consequences of deleting the α2δ auxiliary subunits

Next, using a battery of mechanical, heat and cold nociceptive tests, we examined the behavior of mice in which we constitutively knocked out α2δ-2, α2δ-3 or α2δ-4. We also examined their responses to exogenous application of pruritogens (itch).

### Acute pain responses (naïve conditions)

**Fig 3:**
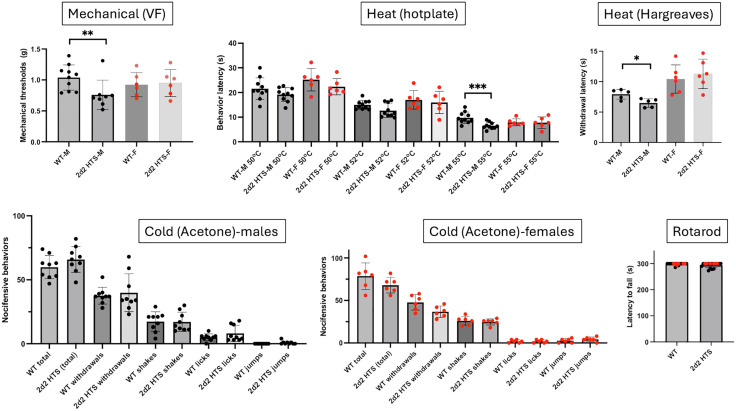
*Cacna2d2* heterozygous male, but not female mice, exhibit hypersensitivity across modalities. We characterized the acute nociceptive responses of mice that are deficient for the *Cacna2d2* gene using a wide range of mechanical (von Frey; VF), heat (hotplate and Hargreaves) and cold (acetone) nociceptive tests. Knocking down *Cacna2d2* led to a reduction in response thresholds to mechanical (VF) and heat (Hargreaves and hotplate at 55^o^C) stimuli; cold responses were unchanged. Global deletion of *Cacna2d2* did not induce any motor deficit in the rotarod assay. Wild-type (WT) and heterozygous (2d2 HTS) mice of the same sex were compared using a Mann Whitney test *p < 0.05; ** p < 0.01; ***p < 0.005. Black circles: males (N = 5-10): Red circles: females (N = 5).

Complete *Cacna2d2* KO mice exhibited severe deficits, both structural (smaller size) as well as behavioral (ataxic, loss of balance), and most died at a young age, which obviously made their study very difficult. On the other hand, as the *Cacna2d2* heterozygous (hts) mice appeared normal, we decided to use the heterozygous mice and analyze the behavioral effects of knocking down (rather than knocking out) the expression levels of *Cacna2d2*. Remarkably, we found that *Cacna2d2* hts male mice were significantly more responsive to mechanical and heat stimuli (Fig 3), but not to a cold stimulus (acetone). In contrast, the nociceptive responses of *Cacna2d2* hts female mice did not differ from their WT counterparts, regardless of the modality.

**Fig 4:**
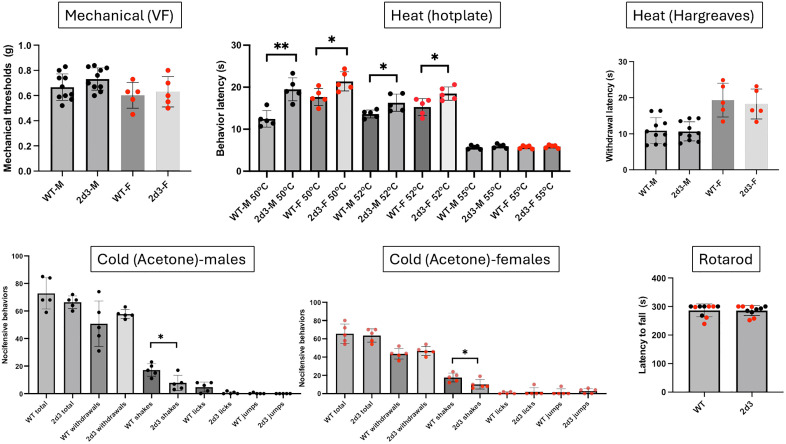
Male and female *Cacna2d3* KO mice exhibit hyposensitivity to thermal stimuli. Here, we used the same mechanical, heat and cold nociceptive tests in mice deficient for the *Cacna2d3* gene and recorded an increase in the latency to respond to a heat stimulus (hotplate at 50^o^C and 52^o^C) and fewer number of shakes in response to a cold (acetone) stimulus. Responses to a mechanical stimulus or to heat in the Hargreaves test were not altered. Global deletion of *Cacna2d3* did not induce any motor deficit in the rotarod assay. Wild-type (WT) and *Cacna2d3* KO mice of the same sex were compared using a Mann Whitney test * p < 0.05; ** p < 0.01. Black circles: males (N = 5-10); Red circles: females (N = 5).

**Fig 5:**
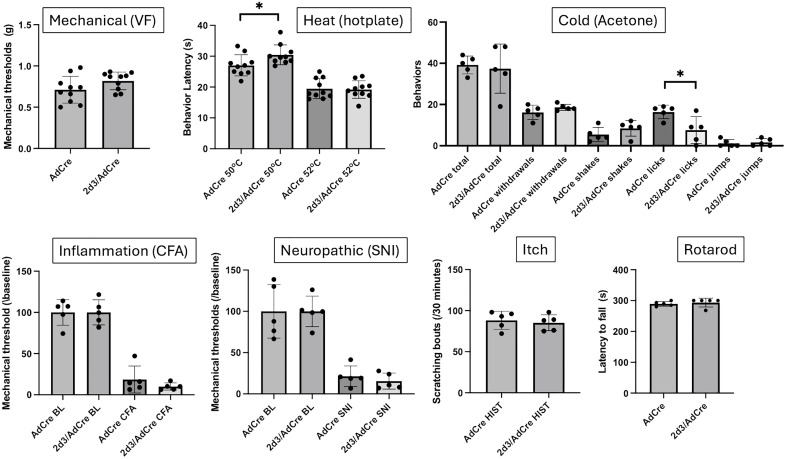
*Cacna2d3* expressed in sensory neurons contributes to the thermal deficits in *Cacna2d3* KO mice. The selective deletion of *Cacna2d3* in DRG neurons recapitulated some of the phenotype recorded in the global *Cacna2d3*, namely decreased responses to thermal (heat and cold) but not mechanical stimuli. On the other hand, the responses of the conditional KO mice in the setting of chronic (inflammatory and neuropathic) pain processing did not differ from the *Advillin*-Cre (AdCre) control mice. Selective deletion of *Cacna2d3* did not induce any motor deficit in the rotarod assay. AdCre and 2d3/AdCre KO mice were compared using a Mann Whitney test * p < 0.05. N = 5-10.

Deleting *Cacna2d3* revealed a very different, indeed an opposite phenotype (Fig 4). The male *Cacna2d3* deficient mice were less responsive to heat when tested on a hotplate at 50^o^C and 52^o^C. Moreover, shakes in responses to a cold stimulus (acetone) were also significantly decreased. In distinct contrast, responsiveness to a von Frey filament or to a heat source (Hargreaves) was not altered. Similarly, *Cacna2d3* female KO mice also exhibited hyposensitivity in the hotplate test at both 50^o^C and 52^o^C as well as in the acetone (cold) test, but not in the Hargreaves or the VF assays. Interestingly, some thermal deficits were recapitulated, to some extent, when *Cacna2d3* was removed selectively from sensory neurons, in double transgenic fl.*Cacna2d3* x *Advillin*-Cre male mice (Fig 5), namely a small but significant increased thermal latency at 50^o^C and fewer number of licks in response to a cold stimulus, compared to the control *Advillin*-Cre mice. Taken together, we conclude that the sensory neuron-expressed *Cacna2d3* likely contributes to the thermal “pain” phenotype recorded in the complete *Cacna2d3* KO mice.

**Fig 6:**
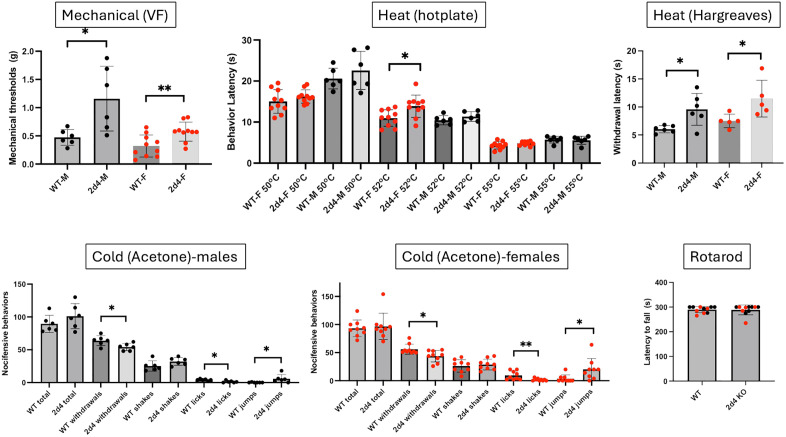
*Cacna2d4* KO mice exhibit hyposensitivity across multiple modalities. Male and female *Cacna2d4* KO mice were less responsive to mechanical, heat and cold stimuli. Global deletion of *Cacna2d4* did not induce any motor deficit in the rotarod assay. Wild-type (WT) and *Cacna2d4* KO mice of the same sex were compared using a Mann Whitney test * p < 0.05; ** p < 0.01. Black circles: males (N = 6); Red circles: females (N = 5-10).

Mice deficient for *Cacna2d4* exhibited hyposensitivity across pain modalities (Fig 6). Specifically, the male *Cacna2d4* KO mice exhibited significantly fewer nociceptive responses to mechanical, heat (HG) or cold stimulation. Female *Cacna2d4* KO mice showed comparable deficits to the KO males. In fact, the deficits were even larger as they also exhibited thermal hypoalgesia in the hotplate assay (at 52^o^C). What partly complicated the test of cold sensitivity is that both male and female *Cacna2d4*KO mice were easily startled by application of the acetone (increased jumps).

Importantly, all WT and knockout male or female mice performed similarly on the rotarod test (Figs 3-6) indicating that the behavioral measures of nociceptive processing that we recorded were not due to motor impairment.

### Nociceptive processing after nerve injury or inflammation

**Fig 7:**
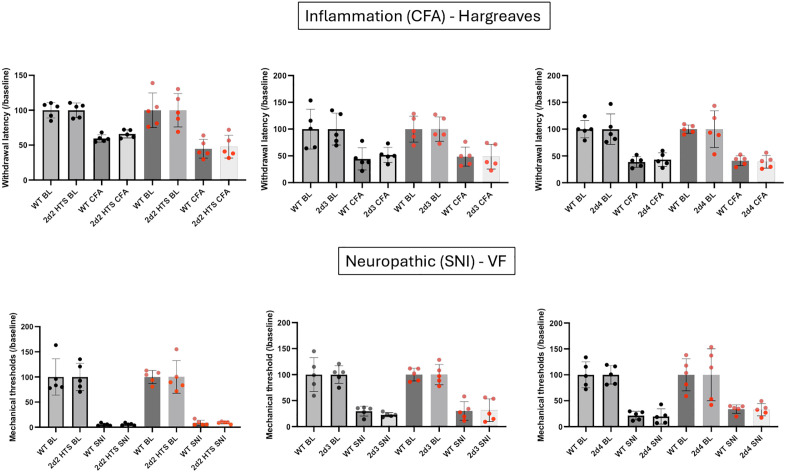
VGCC auxiliary α2δ subtypes do not contribute to chronic pain. In contrast to acute nociception, mice deficient for *Cacna2d2*, *Cacna2d3* or *Cacna2d4* exhibit similar responsiveness to thermal (Hargreaves) or mechanical (VF) stimuli in models of chronic, inflammatory (CFA) or neuropathic (SNI) pain. Wild-type (WT) and KO mice of the same sex were compared using a Mann Whitney test. Black circles: males (N = 5) Red circles: females (N = 5).

Surprisingly, although we recorded many changes in tests of acute pain processing in most of the α2δ KO mice, we found no differences between WT and KO mice following CFA-induced inflammation or SNI (Fig 7), regardless of sex. This result was true for both mechanical (SNI) and thermal (CFA) assays. We conclude that the α2δ auxiliary subunits contribute to acute but not to chronic pain processing, despite their expression levels being altered after peripheral nerve injury.

### Pruritogen-evoked itch

**Fig 8:**
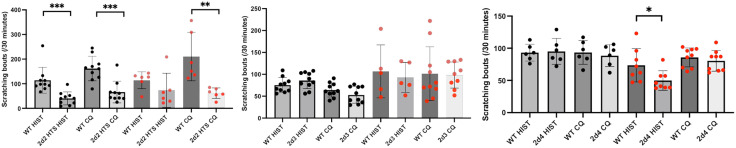
α2δ2 and α2δ4, but not α2δ3, contribute to itch processing. Scratching bouts induced by a nape of the neck injection of histamine (HIST) or chloroquine (CQ) were significantly reduced in mice deficient for *Cacna2d2*. Pruritoception was also decreased in female, but not male *Cacna2d4* KO mice in response to histamine, but not to chloroquine. On the other hand, deletion of *Cacna2d3* had no significant impact on acute itch processing. Wild-type (WT) and KO mice of the same sex were compared using a Mann Whitney test * p < 0.05, ** p < 0.01, ***p < 0.005. Black circles: males (N = 5–10); Red circles: females (N = 5–10).

Interestingly, although the *Cacna2d2* hts mice were hypersensitive across pain modalities, their response to pruritogens (scratching bouts) decreased significantly compared to their WT littermates (Fig 8), regardless of sex. In contrast, pruritogen-evoked scratching in the *Cacna2d3* KO mice did not differ from their WT littermates. On the other hand, we recorded decreased scratching in *Cacna2d4* KO female (but not male) mice, and only in response to histamine; chloroquine induced comparable levels of scratching regardless of genotype.

### 3) Activation of microglia in *Cacna2d2* heterozygous mice

**Fig 9:**
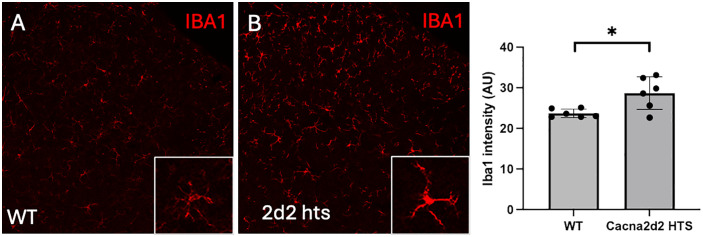
Anatomical changes in α2δ2 subunit mutant mice. The immunoreactivity for Iba1, a microglia marker, was significantly increased in the spinal cord of *Cacna2d2* heterozygous mice, compared to their WT littermates. The spinal cord levels of Iba1 in naïve (WT) and *Cacna2d2* heterozygous mice were quantified using image J and values represent mean intensity fluorescence in arbitrary units (AU); N = 6; Student’s t-test * p < 0.05).

As the complete ablation of *Cacna2d2* induces severe deficits, we also investigated whether the hypersensitivity that we recorded in the hts mutant mice was paralleled by anatomical changes in sensory neurons. Somewhat unexpectedly, we found that the expression of Iba1, a protein marker of microglia, was significantly increased at baseline in the spinal cord dorsal horn of the *Cacna2d2* hts mice (Fig 9). Conceivably this microglial “activation” contributes to the baseline hypersensitivity recorded in these mice [33].

## Discussion

Although functional VGCCs are composed of one of four auxiliary α2δ subunits, unclear is whether there is a redundant or selective functional contribution of the different subunits to pain processing. As the selective deletions led to different, and in some cases opposite pain and itch phenotypes, our findings suggest that the α2δ subunits are not redundant in their contribution. Interestingly, and strikingly different from the profound consequence of α2δ1 deletion, we found that deficits following knockout of α2δ2, 3 or 4 were limited to baseline indices of pain and itch processing. Based on these analyses, we conclude that these α2δ subunits of VGCCs contribute to acute but not chronic pain. The mechanisms underlying these differences remain however to be determined.

Although VGCCs are expressed ubiquitously in all excitable cells [9], the α2δ auxiliary subunits are expressed in a tissue-specific manner [14,34,35]. Thus, tissue and/or cellular localization may be an important factor. For example, we found that the α2δ isoforms have very different patterns of expression in sensory neurons. Of note, both the *Cacna2d2* and *Cacna2d3* are expressed at relatively high baseline levels in primary sensory neurons, but in largely non-overlapping subsets. *Cacna2d2* associated predominantly with unmyelinated neurons (presumably “nociceptors”) and *Cacna2d3* with myelinated neurons (the majority of which are activated by non-noxious mechanical stimuli). Not surprisingly, therefore, deletion of *Cacna2d2* and *Cacna2d3* led to very different nociception outcomes. However, the resultant phenotypes would not have been predicted from their expression patterns, namely hypersensitivity and hyposensitivity, respectively.

We recognize that because the mice that we studied were complete or partial KOs, some of the differences may be attributed to deletion of the genes in the spinal cord or at higher brain levels. In fact, *Cacna2d2* is expressed in many GABAergic neurons in the CNS [14] suggesting a role in modulation or activation of central inhibitory synapses. It follows that knockdown of *Cacna2d2* would reduce calcium currents from α2δ2-containing VGCCs in GABAergic synapses, which could lead to generalized hyperexcitability. Consistent with this view, *Cacna2d2* mutant mice have an epileptic/seizure phenotype. As *Cacna2d2* also predominates in inhibitory neuron subsets in the spinal cord [36], a similar loss of inhibitory tone would likely result in increased nociceptive processing, typical of many neuropathic pain models that arise from loss of Gabaergic inhibitory controls [37]. In fact, we found increased spinal cord Iba1 mRNA and protein immunostaining in microglia of the *Cacna2d2* hts mice, a hallmark of neuropathic pain/hypersensitivity [23,33,38,39].

A previous study reported reduced heat pain processing in Cacna2d3-deficient mice [19] and because these authors did not detect expression of the gene or protein in sensory neurons, they concluded that the phenotype arose from a brain deficit. Interestingly, in their imaging studies, they described a reduction in transmission of “pain” messages from thalamic to cortical areas. A subsequent study reported that loss of *Cacna2d3* induces anatomical alterations in the mutant brain [18] and that noxious stimuli induce less activity (Fos) in pain-relevant neurons of the CNS, all features that can lead to hyposensitivity. Surprisingly, Neely et al did not detect Cacna2d1 in sensory neurons, which contrasts with our own and many other reports [11,28,31,32]. Furthermore, we found a similar phenotype after selective deletion of Cacna2d3 in sensory neurons. Taken together, we conclude that there can be both a sensory neuron and supraspinal contribution to the pain phenotype of the Cacna2d3 knockout mice.

Neely et al also found reduced CFA-induced heat hypersensitivity in the Cacna2d3 mutant mice. In contrast to the deficits in acute heat and cold pain processing in the mutant mice, somewhat unexpectedly, and contrasting the Neely et al report [19], we found that *Cacna2d3* KO mice developed significant post-CFA heat hypersensitivity, equivalent to that of naïve mice. We conclude that CFA can induce the hypersensitivity through Cacna2d3 independent pathways and clearly can overcome the dampening effect observed in the knockout. It is however possible that we missed a transient reduction of hypersensitivity that may have occurred before our testing day (we only tested at day 3 which is when inflammation peaks). Indeed, Neely reported an antihyperalgesic effect that peaked at day 1 (post-CFA) but that rapidly decreased and was no longer significant by day 4–5. Based on our findings, however, we conclude that α2δ3-containing VGCCs are unquestionably contributors to nociceptive processing and that selectively disrupting their activity in DRG neurons may be sufficient to induce hyposensitivity and therefore constitute a promising therapeutic target.

As the *Cacna2d4* mRNA expression was very low in DRG neurons at baseline, the mechanisms underlying decreased nociception in the *Cacna2d4* mutant mice is also interesting, but puzzling. Presumably the deficits that we recorded in the mutant mouse arise from alterations in the CNS. A caveat to this conclusion is that expression of *Cacna2d4* is also very low throughout the brain [40], being mostly detected in non-neuronal peripheral tissues.

Interestingly, the α2δ subunits have not only different patterns of expression in the DRG at baseline but are also differentially modulated by nerve injury. Thus, *Cacna2d2* and *Cacna2d3* were downregulated by nerve injury. In contrast, *Cacna2d4,* which is barely detectable at baseline, was significantly increased after injury. However, as none of the mutant mice exhibited altered behavioral changes in nociceptive processing in a neuropathic pain setting, the consequences of these nerve injury-induced transcriptional changes are unclear and remain to be explored. At the very least, we conclude that the changes were not sufficient to significantly impact the mutant mice’s pain behavior. It is also possible that in the setting of chronic pain, the loss of activity from one α2δ subunit is compensated by other genes with overlapping functions. Interestingly, we note that deleting *Cacna2d3* or *Cacna2d4* led to similar phenotypes (i.e., decreased pain responses) suggesting that these 2 subunits may be redundant in their function and/or engage similar CNS pathways. Clearly, the analysis of double and triple KOs would shed some light on whether some of the recorded phenotypes can be rescued (or perhaps worsen) by other mutations.

Calcium channels also contribute to itch processing. For example, Ca_v_3 (T type) channels, which control mechanical thresholds [31,41] also contribute to acute [42,43] and chronic [44] itch. Although T type calcium channels are not associated with auxiliary subunits, there is indirect evidence for a contribution of α2δ-containing VGCCs to pruritus. For example, gabapentin has anti-pruritic effects in both clinical [45,46] and pre-clinical [47,48] settings, indicating that at least α2δ1-containing VGCCs are engaged. We found that the α2δ2 and α2δ4 isoforms also modulate pruritoception. Indeed, scratching-induced by pruritogens was significantly decreased in both the *Cacna2d2* and *Cacna2d4* mutant mice. Although pain and itch are distinct percepts, they are triggered by subsets of molecularly comparable DRG neurons (TRPV1+), and in fact we found that *Cacna2d2* is expressed in peptidergic sensory neurons, which likely include a pruritic subset. Intriguingly, however, *Cacna2d2* hts mice showed an inverse correlation between pain and itch; they were hypersensitive across multiple pain modalities, but their responses to exogenous pruritogens was decreased. Conceivably, these differences reflect the circuits engaged at the level of the spinal cord by algogenic and pruritogenic Cacna2d2-expressing unmyelinated afferents.

Genetic compensation, whereby the loss of one gene may be compensated by another with overlapping functions, can occur during embryonic development [49]. For this reason, we cannot exclude the possibility of transcriptional adaptation in some of the α2δ mutants. On the other hand, previous analyses of selective α2δ mutants revealed specific deficits that were not compensated by other α2δ isoforms and were unrelated to calcium channel function. For example, α2δ1 mutant mice have dysfunctional cardiomyocytes [50]. Ablation of α2δ2 results in abnormal Purkinje cell dendritic trees [51] as well as other anatomical abnormalities (homozygous mutant mice have smaller size). Mice deficient for α2δ3 exhibit aberrant auditory nerve fiber projections to the cochlear nucleus [52] and α2δ4 KO mice (as well as humans) have abnormal rod and cone photoreceptor synapses [53]. It is, therefore, possible that many of these structural changes impacted the physiological responses of the different isoform mutant mice, including perhaps their behavioral measures of pain and itch processing.

## Conclusions

Here we showed that knocking down the expression of the α2δ subunits of VGCCs has significant impact on pain and itch transmission and that the direction of the change is subunit dependent. Given the alterations in injury-induced expression of these subunits in DRG neurons, it is surprising that the consequence of deleting the subunits was limited to behavior in naïve conditions, i.e., in the absence of injury. Conceivably, in injury settings, in which major transcriptional and translational changes occur, loss of the contribution from a particular α2δ subunit, other than α2δ1, is compensated for by other genes with overlapping functions.

## Supporting information

S1 Fig Spinal cord expression of VGCCs. *In situ* hybridization using selective probes for *Cacna2d1*, *Cacna2d2*, *Cacna2d3* and *Cacna2d4* revealed that *Cacna2d1*, *Cacna2d2* and *Cacna2d3* are expressed in both the dorsal (DH) and ventral (VH) horns of the lumbar spinal cord. *Cacna2d4* levels were very low to undetectable in both DH and VH.
(TIF)

S1 Data Minimal Data Set. (XLSX)
