# Supplementary figures and images for: Differential contribution of α2δ auxiliary subunits of voltage-gated calcium channels in mouse models of pain and itch
Source: PLoS One. 2025 Dec 2;20(12):e0337701. doi: 10.1371/journal.pone.0337701 (PMC12671734; doi:10.1371/journal.pone.0337701)

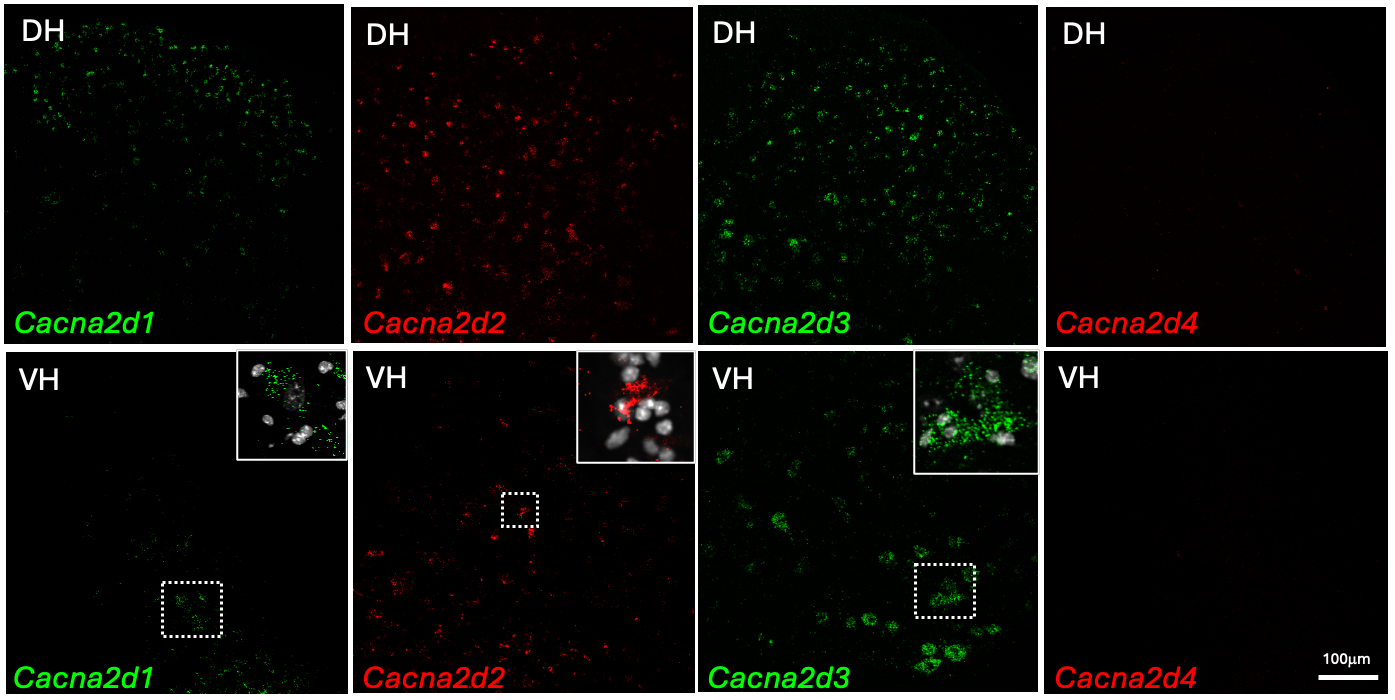

Supplement: S1 Fig — In situ hybridization using selective probes for Cacna2d1, Cacna2d2, Cacna2d3 and Cacna2d4 revealed that Cacna2d1, Cacna2d2 and Cacna2d3 are expressed in both the dorsal (DH) and ventral (VH) horns of the lumbar spinal cord. Cacna2d4 levels were very low to undetectable in both DH and VH. (TIF) [file pone.0337701.s001.tif]
